# Low CT-derived vertebral attenuation as a prognostic factor in hepatocellular carcinoma patients treated with immune checkpoint inhibitors

**DOI:** 10.3389/fonc.2026.1971063

**Published:** 2026-10-07

**Authors:** Shanshan Jiang, Yi Ren, Yuyang Wang, Yi Li, Lianwei Miao, Weiwei Liu, Guofeng Zhou

**Affiliations:** 1 Department of Radiology, Union Hospital, Tongji Medical College, Huazhong University of Science and Technology, Wuhan, China; 2 Hubei Provincial Clinical Research Center for Precision Radiology & Interventional Medicine, Wuhan, China; 3 Hubei Key Laboratory of Molecular Imaging, Wuhan, China

**Keywords:** CT-derived vertebral attenuation, hepatocellular carcinoma, immune checkpoint inhibitors, immunotherapy, prognosis

## Abstract

**Background:**

Computed tomography (CT)-derived vertebral attenuation has been recognized as an indicator of prognosis for patients undergoing tumor removal, liver transplant, or transcatheter arterial chemoembolization (TACE) therapy, but similar research has not been reported in hepatocellular carcinoma (HCC) patients treated with immune checkpoint inhibitors (ICIs). This study aims to explore the prognostic value of CT-derived vertebral attenuation in HCC patients treated with ICIs.

**Methods:**

This retrospective study included HCC patients who received ICI treatment at Wuhan Union Hospital between July 2020 and March 2024. CT-derived vertebral attenuation was assessed by measuring mean Hounsfield unit (HU) values in the center of the first lumbar vertebra (L1) on non-contrast CT scans. Based on the optimal cutoff value derived from X-tile software, patients were classified into the CT-derived L1 attenuation-low group (≤100 HU) and the CT-derived L1 attenuation-high group (>100 HU). A comparison of the baseline characteristics of the two groups was made, followed by a propensity score matching (PSM) analysis to balance the variables. The objective response rate (ORR) and disease control rate (DCR) were calculated for both groups, and Kaplan-Meier survival curves and Cox regression models were used to analyze overall survival (OS) and progression-free survival (PFS).

**Results:**

The cohort consisted of 357 patients, divided into the L1 attenuation-low group (n = 112) and the L1 attenuation-high group (n = 245). Among all patients, 30.0% (107/357) achieved complete or partial response. After PSM analysis, both groups contained 91 patients. The L1 attenuation-low group had shorter OS (hazard ratio [HR], 1.73; 95% confidence interval [CI], 1.15–2.60; P = 0.008) and PFS (HR, 1.47; 95% CI, 1.04–2.08; P = 0.030) than the L1 attenuation-high group.

**Conclusion:**

CT-derived L1 attenuation can stratify survival risk for HCC patients undergoing ICI treatment.

## Introduction

Hepatocellular carcinoma (HCC) is the predominant form of primary liver cancer and is a major factor in mortality linked to cancer globally (1). Based on worldwide epidemiological statistics from 2020, HCC represents 4.69% of all cancer cases and 8.34% of deaths related to cancer (2, 3). Predicting the prognosis of HCC patients is crucial for formulating accurate treatment strategies (4, 5). Current treatment options for HCC include surgery, radiofrequency ablation, transcatheter arterial chemoembolization (TACE), radiotherapy, and chemotherapy; however, the prognosis for advanced-stage patients remains poor (6, 7). Over the past decade, the emergence of immune checkpoint inhibitors (ICIs) has revolutionized anti-tumor therapy (8). ICIs targeting programmed cell death protein 1 (PD-1), programmed death-ligand 1 (PD-L1), and cytotoxic T-lymphocyte-associated protein 4 (CTLA-4) can inhibit the activity of immune checkpoint proteins, thereby reactivating anti-tumor activity. Studies suggest that this immunotherapy is particularly effective for patients with advanced disease, those who are inoperable, and those who have received radiofrequency ablation (9, 10). Nevertheless, a significant proportion of patients exhibit minimal or no response to immunotherapy. This underscores the critical need for reliable prognostic biomarkers to identify candidates eligible for ICI therapy.

Malignant tumors, as wasting diseases, lead to a significantly higher prevalence of bone loss and muscle wasting compared with individuals without cancer (11, 12). The quality of life is negatively impacted by these long-term musculoskeletal conditions, which also contribute to an unfavorable prognosis. We have earlier shown that there is a strong association between sarcopenia and poor prognosis in HCC patients (13), but the correlation between osteopenia and HCC prognosis, particularly in patients receiving immunotherapy, remains less explored. T-scores obtained using dual-energy X-ray absorptiometry (DXA) are considered the gold standard for the diagnosis of osteoporosis (14). Recent studies have suggested that vertebral attenuation derived from computed tomography (CT) correlates with DXA-derived T-scores and has been widely used to assess preoperative osteopenia in patients with gastrointestinal malignancies (15). Some studies have reported that CT-derived vertebral attenuation can stratify the clinical outcomes of lung cancer patients receiving immunotherapy (16). According to previous studies, low pre-transplant CT-derived attenuation of the first lumbar vertebra (L1) is considered an independent factor affecting mortality (17, 18). Additionally, for HCC patients having resection, CT-derived L1 attenuation serves as a robust predictor of survival (19). In a preliminary study, Zheng et al. (20) analyzed the role of CT-derived L1 attenuation alterations during TACE treatment as a possible risk factor. Müller et al. (21) concluded that CT-derived L1 attenuation is an independent predictor of survival in elderly HCC patients receiving TACE treatment. Similar findings have also been reported in other cancers such as breast cancer and digestive cancers (15, 22). Consequently, for HCC patients undergoing ICI treatment, CT-derived vertebral attenuation measurement, as a quantitative indicator of bone health, may similarly play a critical role in prognosis prediction.

In this study, our aim is to assess the influence of CT-derived vertebral attenuation on the outcomes for HCC patients undergoing treatment with ICIs. First, we analyzed the characteristics of 357 patients and used propensity score matching (PSM) analysis to improve baseline covariate balance. Second, we analyzed overall survival (OS) and progression-free survival (PFS) based on CT-derived vertebral attenuation. Finally, we analyzed the prognostic factors related to OS and PFS utilizing Cox regression analysis.

## Materials and methods

Approval for this retrospective cohort study was obtained from the local ethics committee and the institutional review board (IRB) at Tongji Medical College, Huazhong University of Science and Technology (IRB No. 0445). The study followed the guidelines of Good Clinical Practice and the Declaration of Helsinki. Informed consent was not required by the ethics committee due to the study’s retrospective design.

### Study design and patient selection

This research was a retrospective study conducted at a single center. A retrospective review of electronic medical records was conducted for patients adhering to the inclusion criteria. The research involved HCC patients who consecutively received ICI therapy at Wuhan Union Hospital between July 2020 and March 2024. Inclusion criteria: (1) Age >18 years; (2) Diagnosis of HCC based on histological or imaging criteria according to the European Association for the Study of the Liver (EASL) standards (1, 23); (3) Barcelona Clinic Liver Cancer (BCLC) stage B or C; (4) CT scan performed before treatment, with L1 used for calculating CT-derived vertebral attenuation. Exclusion criteria: (1) Clinical or imaging data that were incomplete or could not be evaluated; (2) Palliative surgery, prior neoadjuvant therapy, radiotherapy, or other therapies; (3) Patients with other concurrent malignant tumors; (4) Patients who were not followed up.

### Procedures

Retrospective information was obtained from the medical records of the patients, including the following: demographic data (age, sex, body mass index (BMI) in kg/m², smoking history, alcohol consumption history, Eastern Cooperative Oncology Group (ECOG) performance status); date of first immunotherapy; date of HCC recurrence and the last follow-up or death; Child-Pugh grade; laboratory data (pre-treatment alpha-fetoprotein (AFP), serum total bilirubin, creatinine, serum albumin, alanine transaminase (ALT), aspartate transaminase (AST), hemoglobin (Hb), neutrophil-to-lymphocyte ratio (NLR), platelet-to-lymphocyte ratio (PLR)); history of hepatitis B virus (HBV) infection and use of antiviral medications; pre-treatment imaging data (number of lesions, size of the largest lesion [cm], lymph node metastasis, cirrhosis, portal vein tumor thrombosis (PVTT)); type of ICIs and treatment regimen; CT-derived vertebral attenuation. Baseline data were obtained from the patients’ initial admission and discharge records.

### Measurement of CT-derived vertebral attenuation

The CT scans were conducted on a 128-section CT scanner (SIEMENS SOMATOM Definition AS+, Siemens Healthcare, Erlangen, Germany) with the same settings. Tube voltage was 120 kilovolts peak (kVp), and the tube current was adjusted automatically. The reconstruction method employed a standard soft convolution kernel, with both slice thickness and interval at 1 mm. The images were analyzed by two radiologists (G.Z. and Y.L.) with no clinical data available to them, who had 31 and 4 years of experience in diagnostic imaging. The CT-derived vertebral attenuation was calculated using the Philips IntelliSpace Portal workstation (version 10.1, Best, the Netherlands). The average CT-derived vertebral attenuation from the two separate radiologists was calculated for further analysis, with any discrepancies settled through discussion. Two weeks later, 20% of the scans were selected and re-evaluated using the same method. We used the intraclass correlation coefficient (ICC) to assess reliability. The intra-observer ICC was 0.96, with a 95% confidence interval (CI) of 0.90–0.97; the inter-observer ICC was 0.90 (95% CI, 0.84–0.93). Following previous methods (17, 24), we calculated CT-derived vertebral attenuation as the mean attenuation in Hounsfield units (HU) in the center of the L1 vertebral body on non-contrast CT scans (**Figure 1**). When measuring CT-derived L1 attenuation, the region of interest (ROI) should not be placed near the posterior venous plexus, focal heterogeneity (such as sclerotic or lytic lesions), or lesions (such as compression fractures or imaging-related artifacts). The optimal CT-derived L1 attenuation threshold was determined using X-tile software version 3.6.1 (Yale University School of Medicine, New Haven, Connecticut, United States) (25) and patients were dichotomized into the L1 attenuation-low and L1 attenuation-high groups. DXA measurements were unavailable for many patients in the HCC cohort because they had not undergone DXA examinations. Therefore, pretreatment CT-derived L1 attenuation was used as an opportunistic imaging surrogate of bone health. To examine its relationship with DXA measurements, a supplementary correlation analysis was conducted in 200 adult patients randomly selected from our institutional medical records who had undergone DXA and had available CT examinations. The association between CT-derived L1 attenuation and DXA-derived T-scores was assessed using Spearman’s rank correlation.

**Figure 1:**
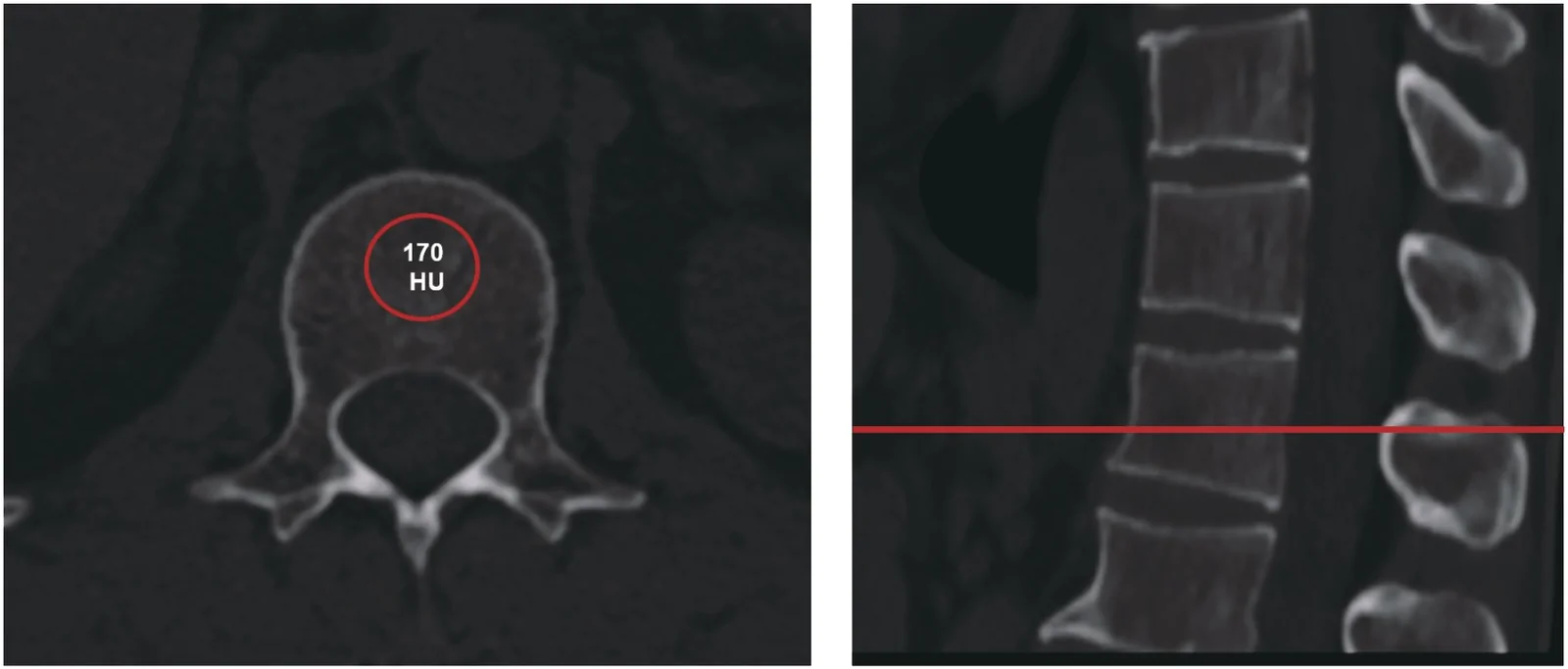
Example for the measurement of the CT-derived L1 attenuation.

### Follow-up and endpoints

Clinical case records, both outpatient and inpatient, along with phone inquiries, were used to obtain follow-up data. The follow-up for all patients continued until July 2024 or until their death. OS was the primary outcome, and PFS was the secondary outcome. OS was defined as the period from the commencement of ICI treatment until the last follow-up or death. The duration of PFS was measured from the initiation of ICI treatment to either tumor progression or death. Treatment response was assessed via CT or magnetic resonance imaging (MRI), applying the modified Response Evaluation Criteria in Solid Tumors (mRECIST) (26). Outcomes were classified as: complete response (CR), partial response (PR), stable disease (SD), or progressive disease (PD). The objective response rate (ORR) comprised CR and PR, whereas the disease control rate (DCR) included CR, PR, and SD.

### Statistical analysis

In this study, continuous variables that followed a normal distribution were expressed as mean ± standard deviation (SD) and compared via the t-test. Variables that did not follow a normal distribution were expressed as median and interquartile range (IQR) and analyzed using the Mann-Whitney U-test. Categorical variables were compared using the chi-square test or Fisher’s exact test. To reduce potential baseline confounding between the L1 attenuation-low and L1 attenuation-high groups, PSM was performed. Propensity scores were estimated using a multivariable logistic regression model, with L1 attenuation group status as the dependent variable and clinically relevant baseline characteristics as covariates, including age, sex, BMI, smoking, drinking, HBV infection status, maximum tumor diameter, tumor number, cirrhosis, lymph node metastasis, PVTT, BCLC stage, Child-Pugh class, ECOG performance status, hepatitis medication history, NLR, PLR, ALT, AST, creatinine, AFP, and albumin-bilirubin (ALBI) grade. Patients were matched using one-to-one nearest-neighbor matching without replacement with a caliper width of 0.1. After matching, baseline balance between groups was assessed using standardized mean differences (SMDs), with an absolute SMD < 0.1 considered indicative of adequate balance. Exploratory Spearman correlation analyses were performed in the original, unmatched cohort to examine the relationships among L1 attenuation group status and selected clinical characteristics, including age, BMI, Hb, Child-Pugh class, ALBI category, cirrhosis, ECOG performance status, NLR, and PLR. Survival curves for PFS and OS were estimated using the Kaplan-Meier method. Univariable and multivariable Cox regression analyses were used to calculate hazard ratios (HRs) and 95% CIs for PFS and OS, taking into account CT-derived L1 attenuation and other clinical factors, to identify independent prognostic factors. The proportional hazards assumption was assessed using Schoenfeld residuals. The multivariable Cox regression analysis included variables with P < 0.1 from the univariable analysis. Exploratory subgroup analyses were conducted after PSM according to clinically relevant covariates. HRs and corresponding 95% CIs were calculated within each subgroup using univariable Cox proportional hazards models. Statistical Package for the Social Sciences (SPSS), version 26.0, from International Business Machines (IBM) and R software version 4.3.0 (R Foundation) were used for statistical analyses. A P value < 0.05 was considered statistically significant.

## Results

### Patient characteristics

This study included 357 HCC patients treated with ICIs (112 in the L1 attenuation-low group and 245 in the L1 attenuation-high group). **Table 1** presents the baseline characteristics of the patients before and after PSM analysis. The median CT-derived L1 attenuation in the entire patient cohort was 130 HU. Compared with the L1 attenuation-high group, patients in the L1 attenuation-low group were older, with an average age of 62 years (P < 0.001), and had a higher proportion of patients in the BCLC stage C (high: 49% vs. low: 63%, P = 0.018) and Child-Pugh grade B (high: 8% vs. low: 17%, P = 0.013). L1 attenuation group status was positively correlated with age (Spearman ρ = 0.346, P < 0.001), indicating that patients in the low-attenuation group tended to be older. Correlations with the remaining clinical characteristics, including BMI, Hb, Child-Pugh class, ALBI category, cirrhosis, ECOG performance status, NLR, and PLR, were weak (all |ρ| ≤ 0.134). Detailed results are presented in **Supplementary Figure 7** and **Supplementary Table 10**. After PSM analysis, each group comprised 91 patients. **Supplementary Table 1** presents the group-specific means or proportions and the SMDs of baseline covariates before and after matching. Overall covariate balance improved after PSM, although residual imbalances remained for drinking, HBV infection, PVTT, BCLC stage, and ECOG performance status, with post-matching absolute SMDs ranging from 0.113 to 0.160. The remaining covariates had post-matching absolute SMDs below 0.1 (**Supplementary Figure 2**).

**Table 1:** Baseline characteristics of patients before and after PSM.

Characteristics	Before PSM			After PSM		
	L1 attenuation-high	L1 attenuation-low	p-value	L1 attenuation-high	L1 attenuation-low	p-value
Patients, n	245	112		91	91	
Age			<0.001			0.710
Mean (± SD)	53 (11)	62 (10)		60 (8)	61 (10)	
Sex, n (%)			0.936			0.661
Female	38 (16%)	17 (15%)		11 (12%)	13 (14%)	
Male	207 (84%)	95 (85%)		80 (88%)	78 (86%)	
BMI (kg/m^2^)			0.289			0.736
Mean (± SD)	22.92 (3.15)	23.32 (3.35)		23.6 (3.4)	23.4 (3.3)	
Smoking, n (%)			0.979			>0.999
No	190 (78%)	87 (78%)		70 (77%)	70 (77%)	
Yes	55 (22%)	25 (22%)		21 (23%)	21 (23%)	
Drinking, n (%)			0.054			0.311
No	221 (90%)	93 (83%)		74 (81%)	79 (87%)	
Yes	24 (10%)	19 (17%)		17 (19%)	12 (13%)	
HBV infection, n (%)			0.127			0.442
No	141 (58%)	74 (66%)		55 (60%)	60 (66%)	
Yes	104 (42%)	38 (34%)		36 (40%)	31 (34%)	
Tumor maximum diameter			0.478			0.622
Median (IQR)	6.8 (4.0, 10.6)	7.4 (4.3, 9.6)		6.3 (3.8, 10.3)	7.5 (4.4, 9.8)	
Tumor number, n (%)			0.824			0.835
≤3	210 (86%)	95 (85%)		78 (86%)	77 (85%)	
>3	35 (14%)	17 (15%)		13 (14%)	14 (15%)	
Cirrhosis, n (%)			0.405			0.550
No	121 (49%)	50 (45%)		38 (42%)	42 (46%)	
Yes	124 (51%)	62 (55%)		53 (58%)	49 (54%)	
Lymph node metastasis, n (%)			0.696			0.720
No	199 (81%)	89 (79%)		70 (77%)	72 (79%)	
Yes	46 (19%)	23 (21%)		21 (23%)	19 (21%)	
PVTT, n (%)			0.150			0.268
No	174 (71%)	71 (63%)		65 (71%)	58 (64%)	
Yes	71 (29%)	41 (37%)		26 (29%)	33 (36%)	
BCLC, n (%)			0.018			0.456
B	125 (51%)	42 (38%)		43 (47%)	38 (42%)	
C	120 (49%)	70 (63%)		48 (53%)	53 (58%)	
Child-Pugh, n (%)			0.013			0.532
A	225 (92%)	93 (83%)		76 (84%)	79 (87%)	
B	20 (8%)	19 (17%)		15 (16%)	12 (13%)	
ECOG, n (%)			0.181			0.415
0	191 (78%)	80 (71%)		67 (74%)	62 (68%)	
≥1	54 (22%)	32 (29%)		24 (26%)	29 (32%)	
Hepatitis drugs, n (%)			<0.001			0.877
No	117 (48%)	75 (67%)		59 (65%)	58 (64%)	
Yes	128 (52%)	37 (33%)		32 (35%)	33 (36%)	
NLR			0.050			0.942
Median (IQR)	2.31 (1.59, 3.17)	2.62 (1.83, 3.66)		2.42 (1.69, 3.50)	2.62 (1.80, 3.67)	
PLR			0.966			0.820
Median (IQR)	118 (88, 159)	111 (93, 159)		115 (79, 156)	114 (97, 159)	
ALT (U/L)			0.140			0.631
Median (IQR)	34 (23, 56)	32 (21, 50)		36 (24, 61)	33 (21, 54)	
AST (U/L)			0.105			0.990
Median (IQR)	43 (30, 75)	40 (28, 63)		46 (31, 70)	42 (28, 66)	
Creatinine (μmol/L)			0.824			0.865
Median (IQR)	67 (57, 76)	64 (56, 73)		67 (58, 77)	64 (57, 73)	
AFP, n (%)			0.487			>0.999
<400	157 (64%)	76 (68%)		60 (66%)	60 (66%)	
≥400	88 (36%)	36 (32%)		31 (34%)	31 (34%)	
ALBI, n (%)			0.114			0.512
1	91 (37%)	32 (29%)		24 (26%)	28 (31%)	
≥2	154 (63%)	80 (71%)		67 (74%)	63 (69%)	

SD, standard deviation; IQR, interquartile range; PSM, propensity score matching; BMI, body mass index; HBV, hepatitis B virus; PVTT, portal vein tumor thrombosis; BCLC, Barcelona Clinic Liver Cancer; ECOG, Eastern Cooperative Oncology Group; NLR, neutrophil-to-lymphocyte ratio; PLR, platelet-to-lymphocyte ratio; ALT, alanine transaminase; AST, aspartate transaminase; ALBI, albumin-bilirubin; AFP, alpha-fetoprotein.

### The cutoff value for CT-derived L1 attenuation

A threshold of 100 HU was used to classify CT-derived L1 attenuation groups based on the X-tile analysis. This cutoff aligns with prior evidence establishing <100 HU as a highly specific diagnostic criterion for osteoporosis in the general population (27). Consequently, patients were dichotomized into the L1 attenuation-low group (≤100 HU) and the L1 attenuation-high group (>100 HU), as illustrated in **Supplementary Figure 1**.

To evaluate the stability of the X-tile-derived cutoff, we performed 1, 000 bootstrap resamples (**Supplementary Figure 3A**). The distribution of the reselected OS cutoffs was centered approximately around 100 HU, and the optimal cutoff estimated from the full cohort was 100.5 HU. The proximity between the bootstrap distribution and the full-cohort estimate supports the internal stability of the cutoff. In the restricted cubic spline analysis (**Supplementary Figure 3B**), vertebral attenuation showed a significant overall association with OS (P = 0.015). The test for nonlinearity was not significant (P = 0.365), suggesting that the association was broadly compatible with a linear pattern across the observed range of attenuation values. The estimated hazard generally decreased with increasing vertebral attenuation, particularly above approximately 100 HU, although the CIs were wider at the extremes of the distribution.

In the supplementary sample of 200 adults, CT-derived L1 attenuation showed a significant positive correlation with DXA-derived T-scores, Spearman’s correlation coefficient was 0.691 (P<0.001) (**Supplementary Figure 4**).

### Tumor response

The tumor response for the L1 attenuation-low and L1 attenuation-high groups before and after PSM analysis is shown in **Supplementary Tables 2**, **3**. Both groups had similar short-term treatment effects overall. Among all patients, 30.0% (107/357) achieved CR or PR. Before PSM analysis, the ORR and DCR for the L1 attenuation-low group were 24.1% and 67.9%, while the ORR and DCR for the L1 attenuation-high group were 32.7% and 76.7%, with no statistically significant difference (ORR, P = 0.171; DCR, P = 0.365). Similarly, after PSM analysis, the ORR (23.1% vs. 30.8%, P = 0.317) and DCR (67.0% vs. 75.8%, P = 0.483) between the two groups were not significantly different. Before PSM analysis, a higher proportion of patients in the L1 attenuation-low group reached PD compared to the L1 attenuation-high group (32.1% vs. 23.3%). This difference remained after PSM analysis (33.0% vs. 24.2%).

### Survival analysis

The follow-up period had a median length of 14.2 months (IQR, 13.0-15.7). The Kaplan-Meier method was used to plot survival curves for PFS and OS. Before PSM analysis, the OS (HR: 1.74 [95% CI: 1.29-2.37]; P < 0.001) and PFS (HR: 1.45 [95% CI: 1.11-1.88]; P = 0.006) in the L1 attenuation-low group were shorter than those in the L1 attenuation-high group (**Figures 2A, B**). Similarly, after PSM analysis, the OS (HR: 1.73 [95% CI: 1.15-2.60]; P = 0.008) and PFS (HR: 1.47 [95% CI: 1.04-2.08]; P = 0.030) in the L1 attenuation-low group were still shorter than those in the L1 attenuation-high group (**Figures 2C, D**).

**Figure 2:**
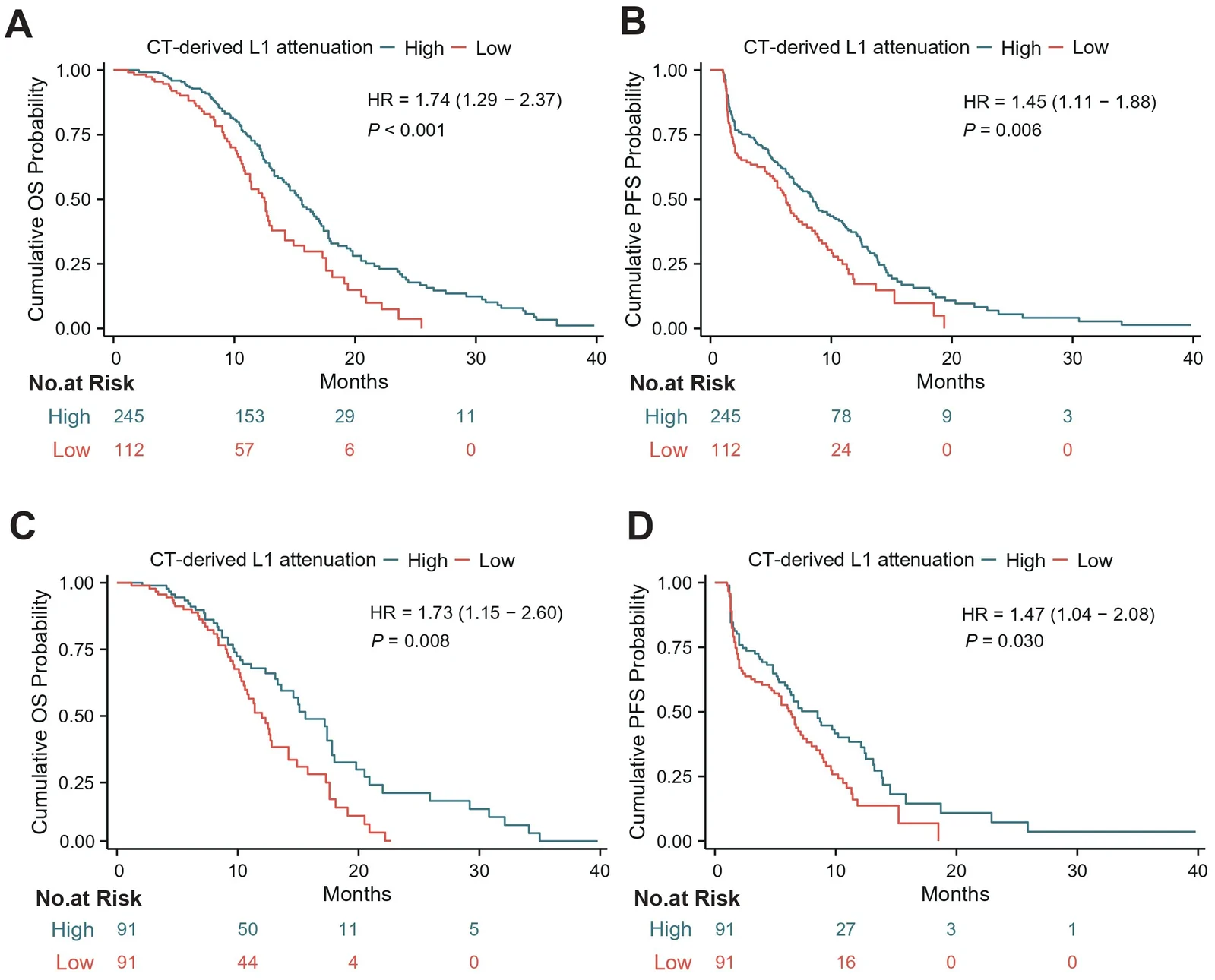
Kaplan-Meier curves for OS **(A)** and PFS **(B)** in the CT-derived L1 attenuation-low group (red) and L1 attenuation-high group (blue) before PSM analysis; Kaplan-Meier curves for OS **(C)** and PFS **(D)** in two groups after PSM analysis. Analyses were conducted using Cox regression. PFS, progression-free survival; OS, overall survival; PSM, propensity score matching.

### Cox regression analysis and subgroup analysis

Before PSM analysis, univariable regression analysis identified CT-derived L1 attenuation, sex, maximum tumor diameter, PVTT, BCLC stage, and AFP as potential prognostic indicators for OS. CT-derived L1 attenuation, age, maximum tumor diameter, PVTT, BCLC stage, PLR, AFP, and creatinine were determined to be potential prognostic indicators for PFS. These covariates were incorporated into the multivariable regression analysis. In multivariable analysis, the L1 attenuation-low group (adjusted HR, 1.800 [95% CI: 1.308-2.476]; P < 0.001), male sex (adjusted HR, 2.344 [95% CI: 1.508-3.644]; P < 0.001), BCLC stage C (adjusted HR, 1.897 [95% CI: 1.252-2.874]; P = 0.003) and AFP ≥ 400 ng/mL (adjusted HR, 1.930 [95% CI: 1.426-2.613]; P < 0.001) were significantly associated with shorter OS (**Supplementary Table 4**). BCLC stage C (adjusted HR, 1.876 [95% CI: 1.337-2.632]; P < 0.001) and the L1 attenuation-low group (adjusted HR, 1.513 [95% CI: 1.112-2.060]; P = 0.008) were significantly associated with shorter PFS (**Supplementary Table 5**).

After PSM analysis, the covariates included in each model are presented in **Tables 2**, **3**. Across the OS and PFS models, the included covariates represented clinically relevant domains: host characteristics (age and sex), tumor burden (maximum tumor diameter and tumor number), macrovascular invasion (PVTT), disease stage (BCLC), tumor biology (AFP), hepatocellular injury (ALT and AST), and renal function (creatinine). CT-derived L1 attenuation status was the exposure of interest. All covariate-specific and global tests based on Schoenfeld residuals yielded P > 0.05. The global P values were 0.4187 for OS and 0.5608 for PFS, indicating no statistically significant evidence of violation of the proportional hazards assumption (**Supplementary Tables 6**, **7**). The L1 attenuation-low group (adjusted HR, 1.759 [95% CI: 1.157-2.675]; P = 0.008), male sex (adjusted HR, 2.202 [95% CI: 1.175-4.128]; P = 0.014) and AFP ≥ 400 ng/mL (adjusted HR, 2.206 [95% CI: 1.427-3.410]; P < 0.001) were significant risk factors for shorter OS (**Table 2**). BCLC stage C (adjusted HR, 1.801 [95% CI: 1.116-2.904]; P = 0.016) and the L1 attenuation-low group (adjusted HR, 1.463 [95% CI: 1.028-2.081]; P = 0.035) were significant risk factors for shorter PFS (**Table 3**).

**Table 2:** Univariable and multivariable Cox proportional hazards analyses for OS after PSM.

Characteristics	Univariable analysis		Multivariable analysis	
	HR (95% CI)	*P* value	HR (95% CI)	*P* value
Age (years)	0.988 (0.965 - 1.012)	0.315		
Sex				
Female	Reference		Reference	
Male	1.860 (1.025 - 3.376)	**0.041**	2.202 (1.175 - 4.128)	**0.014**
Drinking				
No	Reference			
Yes	0.694 (0.393 - 1.224)	0.207		
HBV infection				
No	Reference			
Yes	0.844 (0.559 - 1.273)	0.418		
Tumor maximum diameter (cm)	1.067 (1.018 - 1.119)	**0.007**	1.015 (0.956 - 1.077)	0.629
Tumor number				
≤3	Reference			
>3	0.910 (0.549 - 1.509)	0.714		
PVTT				
No	Reference		Reference	
Yes	2.041 (1.377 - 3.026)	**< 0.001**	1.455 (0.876 - 2.417)	0.148
BCLC stage				
B	Reference		Reference	
C	2.083 (1.326 - 3.271)	**0.001**	1.287 (0.706 - 2.347)	0.411
Child-Pugh				
A	Reference			
B	0.821 (0.436 - 1.547)	0.542		
ECOG				
0	Reference			
≥1	1.149 (0.749 - 1.764)	0.525		
Hepatitis drugs				
No	Reference			
Yes	1.086 (0.700 - 1.686)	0.712		
NLR	0.995 (0.922 - 1.074)	0.905		
PLR	1.000 (0.997 - 1.002)	0.787		
ALT (U/L)	1.004 (1.000 - 1.007)	**0.025**	1.004 (0.997 - 1.011)	0.251
AST (U/L)	1.004 (1.001 - 1.007)	**0.006**	0.999 (0.992 - 1.006)	0.759
Creatinine (μmol/L)	0.997 (0.985 - 1.010)	0.675		
ALBI				
1	Reference			
≥2	1.466 (0.925 - 2.322)	0.103		
AFP (ng/mL)				
<400	Reference		Reference	
≥400	2.484 (1.657 - 3.723)	**< 0.001**	2.206 (1.427 - 3.410)	**< 0.001**
Group				
L1 attenuation-high	Reference		Reference	
L1 attenuation-low	1.730 (1.150 - 2.601)	**0.008**	1.759 (1.157 - 2.675)	**0.008**

OS, overall survival; PSM, propensity score matching; HR, hazard ratio; CI, confidence interval; HBV, hepatitis B virus; PVTT, portal vein tumor thrombosis; BCLC, Barcelona Clinic Liver Cancer; ECOG, Eastern Cooperative Oncology Group; NLR, neutrophil-to-lymphocyte ratio; PLR, platelet-to-lymphocyte ratio; ALT, alanine transaminase; AST, aspartate transaminase; ALBI, albumin-bilirubin; AFP, alpha-fetoprotein. Bold P values indicate statistical significance (P < 0.05).

**Table 3:** Univariable and multivariable Cox proportional hazards analyses for PFS after PSM.

Characteristics	Univariable analysis		Multivariable analysis	
	HR (95% CI)	*P* value	HR (95% CI)	*P* value
Age (years)	0.974 (0.957 - 0.991)	**0.004**	0.994 (0.974 - 1.015)	0.580
Sex				
Female	Reference		Reference	
Male	0.578 (0.365 - 0.916)	**0.020**	0.742 (0.440 - 1.252)	0.264
Drinking				
No	Reference			
Yes	1.006 (0.630 - 1.605)	0.981		
HBV infection				
No	Reference			
Yes	1.284 (0.899 - 1.834)	0.169		
Tumor maximum diameter (cm)	1.067 (1.024 - 1.113)	**0.002**	1.031 (0.982 - 1.082)	0.216
Tumor number				
≤3	Reference		Reference	
>3	1.808 (1.156 - 2.828)	**0.009**	1.538 (0.940 - 2.517)	0.087
PVTT				
No	Reference		Reference	
Yes	2.041 (1.435 - 2.902)	**< 0.001**	1.140 (0.726 - 1.791)	0.569
BCLC stage				
B	Reference		Reference	
C	2.494 (1.730 - 3.595)	**< 0.001**	1.801 (1.116 - 2.904)	**0.016**
Child-Pugh				
A	Reference			
B	0.784 (0.470 - 1.308)	0.351		
ECOG				
0	Reference			
≥1	1.004 (0.693 - 1.455)	0.984		
Hepatitis drugs				
No	Reference			
Yes	0.804 (0.553 - 1.170)	0.254		
NLR	0.991 (0.927 - 1.059)	0.784		
PLR	1.001 (0.998 - 1.003)	0.616		
ALT (U/L)	1.000 (0.997 - 1.003)	0.973		
AST (U/L)	1.001 (0.998 - 1.004)	0.488		
Creatinine (μmol/L)	0.983 (0.971 - 0.994)	**0.004**	0.993 (0.981 - 1.006)	0.285
ALBI				
1	Reference			
≥2	1.085 (0.741 - 1.590)	0.674		
AFP (ng/mL)				
<400	Reference		Reference	
≥400	1.837 (1.285 - 2.626)	**< 0.001**	1.343 (0.919 - 1.961)	0.127
Group				
L1 attenuation-high	Reference		Reference	
L1 attenuation-low	1.471 (1.039 - 2.083)	**0.030**	1.463 (1.028 - 2.081)	**0.035**

PFS, progression-free survival; PSM, propensity score matching; HR, hazard ratio; CI, confidence interval; HBV, hepatitis B virus; PVTT, portal vein tumor thrombosis; BCLC, Barcelona Clinic Liver Cancer; ECOG, Eastern Cooperative Oncology Group; NLR, neutrophil-to-lymphocyte ratio; PLR, platelet-to-lymphocyte ratio; ALT, alanine transaminase; AST, aspartate transaminase; ALBI, albumin-bilirubin; AFP, alpha-fetoprotein. Bold P values indicate statistical significance (P < 0.05).

Exploratory subgroup analyses based on clinically relevant covariates were further performed among patients after PSM. HRs with 95% CIs were estimated for each subgroup using univariable Cox proportional hazards models. The associations between L1 attenuation status and both PFS and OS remained generally consistent across the examined subgroups. Subgroup analyses are presented in **Figures 3**, **4**. For PFS, no statistically significant interactions were detected between CT-derived L1 attenuation status and the examined subgroup variables (all P for interaction ≥ 0.120). For OS, a nominally significant interaction was observed between CT-derived L1 attenuation status and ALBI grade (P for interaction = 0.025). The HR for low versus high CT-derived L1 attenuation was 3.06 (95% CI, 1.30–7.21) among patients with ALBI grade 1 and 1.25 (95% CI, 0.78–2.00) among those with ALBI grade ≥ 2. No statistically significant interactions were detected for the remaining subgroup variables (all P for interaction ≥ 0.132).

**Figure 3:**
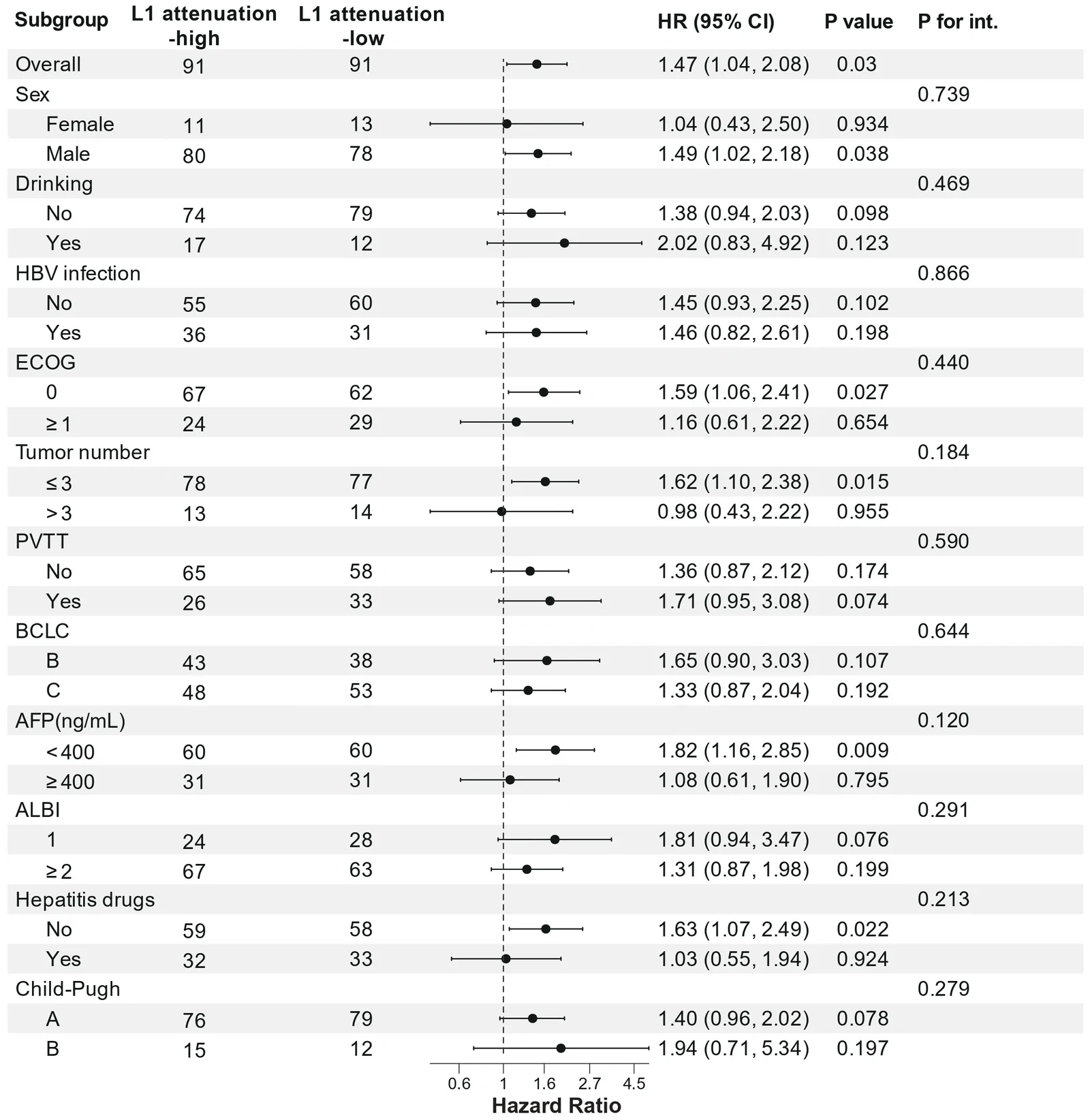
Forest plot of subgroup analysis in progression-free survival between the CT-derived L1 attenuation-low group and L1 attenuation-high group. Dashed line indicates Hazard ratio of 1. HR, hazard ratio; CI, confidence interval; HBV, hepatitis B virus; ECOG, Eastern Cooperative Oncology Group; PVTT, portal vein tumor thrombosis; BCLC, Barcelona Clinic Liver Cancer; AFP, alpha-fetoprotein; ALBI, albumin-bilirubin.

**Figure 4:**
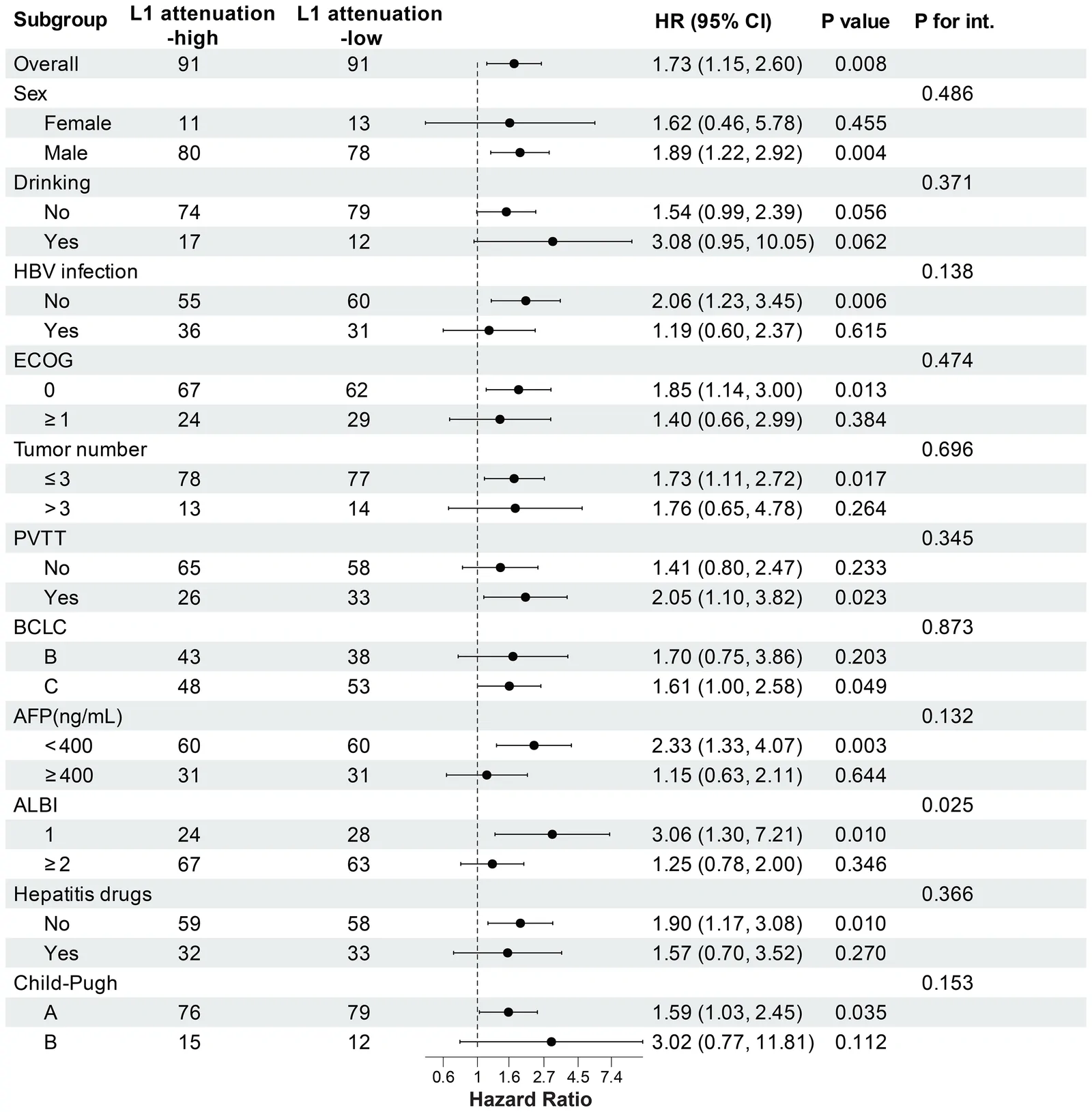
Forest plot of subgroup analysis in overall survival between the CT-derived L1 attenuation-low group and L1 attenuation-high group. Dashed line indicates Hazard ratio of 1. HR, hazard ratio; CI, confidence interval; HBV, hepatitis B virus; ECOG, Eastern Cooperative Oncology Group; PVTT, portal vein tumor thrombosis; BCLC, Barcelona Clinic Liver Cancer; AFP, alpha-fetoprotein; ALBI, albumin-bilirubin.

We performed additional analyses evaluating the available information on treatment regimen and ICI class (PD-1 versus PD-L1 inhibitors). After adjustment, low CT-derived L1 attenuation remained associated with shorter PFS (adjusted HR, 1.458; 95% CI, 1.007–2.110; P = 0.046), consistent with the direction and magnitude of the original estimate (**Supplementary Table 9**). In the updated OS model, low CT-derived L1 attenuation also remained associated with shorter survival (adjusted HR, 1.839; 95% CI, 1.193–2.833; P = 0.006; **Supplementary Table 8**). We additionally conducted exploratory subgroup analyses and likelihood-ratio tests for interaction. No statistically significant interactions were detected between L1 attenuation status and treatment regimen (OS, P = 0.671; PFS, P = 0.341) or ICI class (OS, P = 0.899; PFS, P = 0.483; **Supplementary Figures 5**, **6**). Multivariable Cox regression analysis of PFS showed that ICIs combined with targeted and interventional therapies were associated with a higher risk of disease progression than ICI alone (adjusted HR = 1.951, 95% CI: 1.088–3.498, P = 0.025). However, the overall test for treatment regimen did not reach statistical significance (P = 0.117).

The subgroup results should be interpreted cautiously because some subgroups had limited sample sizes, particularly the female subgroup and the PD-L1 inhibitor subgroup, where the statistical power was insufficient for definitive assessment of subgroup-specific associations.

## Discussion

To the best of our knowledge, this research is the first study to explore the correlation between CT-derived L1 attenuation and both short-term and long-term survival outcomes in HCC patients undergoing ICI treatment. The cutoff value was determined using X-tile software. PSM (caliper width = 0.1) reduced baseline imbalances between groups, although some residual imbalances remained; multivariable Cox regression analysis identified CT-derived L1 attenuation as an independent prognostic factor. Our results show that both before and after PSM, low CT-derived L1 attenuation is significantly associated with shorter OS and PFS. Therefore, assessing CT-derived L1 attenuation could provide additional information for clinical decision-making regarding HCC patients initiating ICI therapy.

Opportunistic assessment of skeletal health using routinely acquired CT scans has increasingly gained attention in patients with chronic liver disease and malignancies. Although DXA remains the reference standard for bone mineral assessment, increasing evidence indicates that CT-derived vertebral attenuation correlates with DXA-based measurements and can serve as an accessible surrogate marker of skeletal health without additional imaging examinations or radiation exposure (24, 28–30). Consistent with this literature, our supplementary analysis of 200 adults demonstrated a significant positive correlation between CT-derived L1 attenuation and DXA-derived T-scores. This finding provides additional support for interpreting vertebral attenuation as an imaging surrogate of skeletal health. We assessed CT-derived L1 attenuation using the average HU at the L1 level, a method also used by several other studies (17). This anatomical landmark is easily available in abdominal and thoracic CT scans. Previous studies have shown that CT-derived vertebral attenuation serves as a prognostic marker in patients undergoing hepatic resection, liver transplantation, or TACE (17–19). Importantly, recent studies have further expanded the application of opportunistic CT-based bone assessment in liver transplantation populations. DXA-referenced analyses in liver transplant recipients have demonstrated the feasibility of vertebral attenuation measurement from contrast-enhanced abdominal CT for bone health evaluation (31). In addition, longitudinal CT studies in patients undergoing liver transplantation, including those with hepatitis B-related liver disease with or without HCC, have shown dynamic and parallel changes in skeletal and muscle compartments after transplantation (32). These findings provide an important imaging-based context for our study and support the potential role of CT-derived vertebral attenuation as a surrogate marker reflecting skeletal health and systemic alterations in patients with liver disease.

Our study demonstrated that lower CT-derived L1 vertebral attenuation was independently associated with shorter OS and PFS, whereas no significant differences were observed in ORR or DCR. ORR and DCR mainly reflect short-term changes in tumor burden and have been widely used as early efficacy endpoints in oncology trials. However, accumulating evidence indicates that these early tumor-based endpoints do not always translate into corresponding improvements in OS, highlighting a potential dissociation between response rates, PFS, and OS (33). This phenomenon has also been observed in randomized immunotherapy trials, where improvements in OS were reported despite limited improvement in objective tumor response measures, suggesting that the clinical benefit of ICIs may extend beyond immediate tumor shrinkage (34, 35). Therefore, the association between lower vertebral attenuation and survival outcomes may reflect host-related vulnerability, impaired physiological reserve, and skeletal health rather than a direct relationship with ICI efficacy.

The biological mechanisms underlying the association between lower CT-derived vertebral attenuation and poor survival remain uncertain. Previous studies have suggested that bone metabolism and immune regulation are closely interconnected through osteoimmunological pathways (36, 37). Immune-derived cytokines, including macrophage colony-stimulating factor (M-CSF), interleukins (ILs), transforming growth factor-beta (TGF-β), and interferon-gamma, participate in osteoclast differentiation and bone remodeling, whereas tumor-derived factors such as parathyroid hormone-related protein (PTHrP), interleukin-1 (IL-1), interleukin-6 (IL-6), and interleukin-8 (IL-8) may disrupt bone homeostasis through enhanced osteoclast activation and signaling mediated by receptor activator of nuclear factor-kappa B (RANK) and receptor activator of nuclear factor-kappa B ligand (RANKL). This pathological shift disrupts the osteoblast-osteoclast equilibrium, activates the RANKL/RANK signaling axis, and promotes bone resorption. Critically, this cascade fosters an immunosuppressive tumor microenvironment (TME), a mechanism implicated in therapy resistance and adverse oncologic outcomes (38, 39). However, the contribution of these mechanisms to CT-derived vertebral attenuation and ICI outcomes in HCC remains hypothetical and requires further validation. In addition, lower CT-derived L1 attenuation may serve as an imaging surrogate of systemic frailty, sarcopenia, impaired nutritional status, or reduced physiological reserve, which may influence long-term survival by affecting treatment tolerance, susceptibility to complications, and subsequent treatment opportunities (40). In our study, the association between CT-derived vertebral attenuation and several clinically relevant factors was further explored. Except for age, which showed a moderate correlation with grouping status, correlations with other important indicators, including BMI, Hb, Child-Pugh classification, ALBI grade, cirrhosis, ECOG performance status, NLR, and PLR, were generally weak. These findings suggest that reduced vertebral attenuation may not simply represent overall frailty or general health deterioration, but may instead capture additional information related to skeletal health.

Clinically, CT-derived vertebral attenuation assessment may help identify patients with potentially increased vulnerability who may benefit from closer surveillance and comprehensive supportive management, including nutritional assessment and optimization of skeletal health (41, 42). It is worth noting that HCC is a heterogeneous disease, so when making decisions, CT-derived L1 attenuation is only one of the various components considered. In multivariable Cox regression analysis, in addition to CT-derived L1 attenuation, AFP ≥ 400 ng/mL and male sex were recognized as independent prognostic indicators for OS, whereas BCLC stage C was found to be an independent prognostic indicator for PFS. AFP, a well-known HCC-related tumor marker, is recognized as a risk factor, with its overexpression reflecting more aggressive tumor biology (43). AFP has been an essential component in traditional scoring methods for anticipating the prognosis of HCC patients treated with TACE (44). Evidence also indicates that AFP is an independent predictor of median OS (45). At the molecular level, AFP is involved in tumor angiogenesis and may influence tumor cell proliferation and tissue growth (43). The matched cohort exhibited male predominance (86.8% vs. 13.2%), consistent with the higher epidemiological incidence of HCC in males, which likely explains the identification of male sex as an independent risk factor in multivariable regression analysis. Further research is needed involving a larger number of female liver cancer patients receiving immunotherapy. According to guidelines from the EASL and the American Association for the Study of Liver Diseases (AASLD), the BCLC classification system serves as a guide for categorizing, determining treatment options, and predicting outcomes of HCC patients. BCLC stage is strongly correlated with prognosis in patients with HCC (1, 46).

There are a few limitations to this study. First, as a retrospective study conducted in a single institution, our results should be validated in multi-center prospective studies. Second, patients who underwent only MRI for baseline assessment were excluded, potentially limiting the sample size and generalizability. Third, CT-derived vertebral attenuation was measured at a single vertebral level and should be considered a surrogate marker of skeletal health rather than a direct assessment of quantitative BMD. Although it has been shown to correlate with DXA-based measurements, single-level attenuation may not fully reflect overall skeletal status. Fourth, the cutoff value used for defining low and high vertebral attenuation was retrospectively determined, and although it was consistent with previously reported thresholds, potential optimism bias and limited generalizability remain possible. Fifth, despite PSM and multivariable adjustment, residual confounding cannot be completely excluded. Sixth, treatment heterogeneity existed because patients received different ICI agents, combination regimens, treatment lines, and subsequent therapies during the study period, which may have influenced survival outcomes. Seventh, although exploratory subgroup analyses were performed, some subgroups had limited sample sizes, particularly female patients, and these findings should be interpreted cautiously and require validation in larger prospective cohorts. In addition, because all CT examinations in this study were performed on a single scanner using a standardized protocol, the measured vertebral attenuation values and the derived 100-HU threshold may be protocol-specific. Variations in scanner calibration, tube voltage, reconstruction algorithm, slice thickness, and contrast-enhancement status may affect HU measurements. Therefore, the applicability of this threshold across different CT scanners and acquisition protocols requires confirmation in external multicenter cohorts, preferably with standardized calibration procedures. Finally, because the cutoff was selected and evaluated in the same cohort, optimism and overfitting cannot be completely excluded. Bootstrap resampling was used to assess internal stability, but independent external validation was not available. Therefore, the 100-HU threshold should be interpreted as an exploratory cutoff rather than a definitively validated clinical threshold. Future multicenter studies should validate this value using standardized CT acquisition and measurement protocols.

## Conclusion

In summary, this study reveals that low CT-derived L1 attenuation is independently associated with poor survival in HCC patients receiving ICI treatment. This finding suggests that assessment of CT-derived L1 attenuation may provide additional information for clinical decision-making for HCC patients initiating ICI therapy.

## Data Availability

The raw data supporting the conclusions of this article will be made available by the authors, without undue reservation.

## Glossary

AASLD: American Association for the Study of Liver Diseases
AFP: Alpha-fetoprotein
ALBI: Albumin-bilirubin
ALT: Alanine transaminase
AST: Aspartate transaminase
BCLC: Barcelona Clinic Liver Cancer
BMD: Bone mineral density
BMI: Body mass index
CI: Confidence interval
CR: Complete response
CT: Computed tomography
CTLA-4: Cytotoxic T-lymphocyte-associated protein 4
DCR: Disease control rate
DXA: Dual-energy X-ray absorptiometry
EASL: European Association for the Study of the Liver
ECOG: Eastern Cooperative Oncology Group
Hb: Hemoglobin
HBV: Hepatitis B virus
HCC: Hepatocellular carcinoma
HR: Hazard ratio
HU: Hounsfield unit
IBM: International Business Machines
ICC: Intraclass correlation coefficient
ICIs: Immune checkpoint inhibitors
IL-1: Interleukin-1
IL-6: Interleukin-6
IL-8: Interleukin-8
ILs: Interleukins
IQR: Interquartile range
IRB: Institutional review board
kVp: Kilovolts peak
L1: First lumbar vertebra
M-CSF: Macrophage colony-stimulating factor
mRECIST: Modified Response Evaluation Criteria in Solid Tumors
MRI: Magnetic resonance imaging
NLR: Neutrophil-to-lymphocyte ratio
ORR: Objective response rate
OS: Overall survival
PD: Progressive disease
PD-1: Programmed cell death protein 1
PD-L1: Programmed death-ligand 1
PFS: Progression-free survival
PLR: Platelet-to-lymphocyte ratio
PR: Partial response
PSM: Propensity score matching
PTHrP: Parathyroid hormone-related protein
PVTT: Portal vein tumor thrombosis
RANK: Receptor activator of nuclear factor-kappa B
RANKL: Receptor activator of nuclear factor-kappa B ligand
ROI: Region of interest
SD: Stable disease
SD: Standard deviation
SMD: Standardized mean difference
SPSS: Statistical Package for the Social Sciences
TACE: Transcatheter arterial chemoembolization
TGF-β: Transforming growth factor-beta
TME: Tumor microenvironment
