# Supplementary material for: Low CT-derived vertebral attenuation as a prognostic factor in hepatocellular carcinoma patients treated with immune checkpoint inhibitors
Source: Front Oncol. 2026 Oct 7;16:1971063. doi: 10.3389/fonc.2026.1971063 (PMC13683168; doi:10.3389/fonc.2026.1971063)
Supplement: Supplementary file 1 [file SupplementaryFile1.docx]

**Supplementary Material**

**Low CT-derived Vertebral Attenuation as a Prognostic Factor in Hepatocellular Carcinoma Patients Treated with Immune Checkpoint Inhibitors**

**Table of Contents**

[Supplementary Tables	3](#_Toc240654042)

[Supplementary Table 1 Baseline characteristics and standardized mean differences between the CT-derived L1 attenuation-low and L1 attenuation-high groups before and after propensity score matching	3](#_Toc240654043)

[Supplementary Table 2 Tumor response in the CT-derived L1 attenuation-low and L1 attenuation-high groups before PSM	5](#_Toc240654044)

[Supplementary Table 3 Tumor response in the CT-derived L1 attenuation-low and L1 attenuation-high groups after PSM	6](#_Toc240654045)

[Supplementary Table 4 Univariate and multivariable Cox proportional hazards analyses for OS before PSM	7](#_Toc240654046)

[Supplementary Table 5 Univariate and multivariable Cox proportional hazards analyses for PFS before PSM	8](#_Toc240654047)

[Supplementary Table 6 Schoenfeld residual tests of the proportional hazards assumption for the multivariable OS model after PSM	9](#_Toc240654048)

[Supplementary Table 7 Schoenfeld residual tests of the proportional hazards assumption for the multivariable PFS model after PSM	10](#_Toc240654049)

[Supplementary Table 8 Univariate and multivariable Cox proportional hazards analyses for overall survival after propensity score matching, including ICI type and treatment regimen.	11](#_Toc240654050)

[Supplementary Table 9 Univariate and multivariable Cox proportional hazards analyses for progression-free survival after propensity score matching, including ICI type and treatment regimen.	13](#_Toc240654051)

[Supplementary Table 10 Correlation coefficients and P values among L1 attenuation group status and clinical characteristics	15](#_Toc240654052)

[Supplementary Figures	16](#_Toc240654053)

[Supplementary Figure 1. The optimal cutoff value for CT-derived L1 attenuation classification was established by the X-tile software. (A) X-tile plots. (B) Histograms.	16](#_Toc240654054)

[Supplementary Figure 2. Love plot showing baseline covariate balance before and after propensity score matching.	17](#_Toc240654055)

[Supplementary Figure 3. Bootstrap assessment (A) of cutoff stability and restricted cubic spline analysis (B) of the association between CT-derived vertebral attenuation and OS.	18](#_Toc240654056)

[Supplementary Figure 4. Scatter plot showing the correlation between CT-derived L1 attenuation and corresponding DXA T-scores.	19](#_Toc240654057)

[Supplementary Figure 5. Forest plot of subgroup analyses for overall survival after propensity score matching, including ICI type and treatment regimen.	20](#_Toc240654058)

[Supplementary Figure 6. Forest plot of subgroup analyses for progression-free survival after propensity score matching, including ICI type and treatment regimen.	21](#_Toc240654059)

[Supplementary Figure 7. Heat map of correlation coefficients between L1 attenuation group status and selected clinical characteristics.	22](#_Toc240654060)

# Supplementary Tables

## Supplementary Table 1 Baseline characteristics and standardized mean differences between the CT-derived L1 attenuation-low and L1 attenuation-high groups before and after propensity score matching

| Variables | Level | Before matching | | |  | After matching | | |
| --- | --- | --- | --- | --- | --- | --- | --- | --- |
|  |  | High | Low | SMD |  | High | Low | SMD |
| n |  | 245 | 112 |  |  | 91 | 91 |  |
| Age (years), mean (SD) |  | 53.43 (10.86) | 62.25 (10.30) | 0.857 |  | 60.36 (8.21) | 60.87 (10.00) | 0.049 |
| Sex (%) | Female | 38 (15.5) | 17 (15.2) | -0.009 |  | 11 (12.1) | 13 (14.3) | 0.061 |
|  | Male | 207 (84.5) | 95 (84.8) | 0.009 |  | 80 (87.9) | 78 (85.7) | -0.061 |
| BMI (kg/m²), mean (SD) |  | 22.92 (3.15) | 23.32 (3.35) | 0.119 |  | 23.61 (3.37) | 23.44 (3.35) | -0.050 |
| Smoking (%) | No | 190 (77.6) | 87 (77.7) | 0.003 |  | 70 (76.9) | 70 (76.9) | 0.000 |
|  | Yes | 55 (22.4) | 25 (22.3) | -0.003 |  | 21 (23.1) | 21 (23.1) | 0.000 |
| Drinking (%) | No | 221 (90.2) | 93 (83.0) | -0.191 |  | 74 (81.3) | 79 (86.8) | 0.146 |
|  | Yes | 24 (9.8) | 19 (17.0) | 0.191 |  | 17 (18.7) | 12 (13.2) | -0.146 |
| HBV infection (%) | No | 141 (57.6) | 74 (66.1) | 0.180 |  | 55 (60.4) | 60 (65.9) | 0.116 |
|  | Yes | 104 (42.4) | 38 (33.9) | -0.180 |  | 36 (39.6) | 31 (34.1) | -0.116 |
| Tumor maximum diameter (cm), mean (SD) |  | 7.65 (4.59) | 7.32 (3.86) | -0.086 |  | 7.11 (4.19) | 7.40 (3.87) | 0.076 |
| Tumor number (%) | ≤3 | 210 (85.7) | 95 (84.8) | -0.025 |  | 78 (85.7) | 77 (84.6) | -0.031 |
|  | >3 | 35 (14.3) | 17 (15.2) | 0.025 |  | 13 (14.3) | 14 (15.4) | 0.031 |
| Cirrhosis (%) | No | 121 (49.4) | 50 (44.6) | -0.095 |  | 38 (41.8) | 42 (46.2) | 0.088 |
|  | Yes | 124 (50.6) | 62 (55.4) | 0.095 |  | 53 (58.2) | 49 (53.8) | -0.088 |
| Lymph node metastasis (%) | No | 199 (81.2) | 89 (79.5) | -0.044 |  | 70 (76.9) | 72 (79.1) | 0.054 |
|  | Yes | 46 (18.8) | 23 (20.5) | 0.044 |  | 21 (23.1) | 19 (20.9) | -0.054 |
| PVTT (%) | No | 174 (71.0) | 71 (63.4) | -0.158 |  | 65 (71.4) | 58 (63.7) | -0.160 |
|  | Yes | 71 (29.0) | 41 (36.6) | 0.158 |  | 26 (28.6) | 33 (36.3) | 0.160 |
| BCLC (%) | B | 125 (51.0) | 42 (37.5) | -0.279 |  | 43 (47.3) | 38 (41.8) | -0.113 |
|  | C | 120 (49.0) | 70 (62.5) | 0.279 |  | 48 (52.7) | 53 (58.2) | 0.113 |
| Child-Pugh (%) | A | 225 (91.8) | 93 (83.0) | -0.234 |  | 76 (83.5) | 79 (86.8) | 0.088 |
|  | B | 20 (8.2) | 19 (17.0) | 0.234 |  | 15 (16.5) | 12 (13.2) | -0.088 |
| ECOG (%) | 0 | 191 (78.0) | 80 (71.4) | -0.145 |  | 67 (73.6) | 62 (68.1) | -0.122 |
|  | ≥1 | 54 (22.0) | 32 (28.6) | 0.145 |  | 24 (26.4) | 29 (31.9) | 0.122 |
| Hepatitis drugs (%) | No | 117 (47.8) | 75 (67.0) | 0.408 |  | 59 (64.8) | 58 (63.7) | -0.023 |
|  | Yes | 128 (52.2) | 37 (33.0) | -0.408 |  | 32 (35.2) | 33 (36.3) | 0.023 |
| NLR, mean (SD) |  | 2.73 (1.89) | 3.34 (3.02) | 0.202 |  | 3.09 (2.31) | 3.07 (2.08) | -0.008 |
| PLR, mean (SD) |  | 135.75 (82.55) | 136.13 (76.06) | 0.005 |  | 132.22 (79.86) | 134.73 (67.94) | 0.033 |
| ALT (U/L), mean (SD) |  | 58.80 (96.34) | 47.19 (51.45) | -0.226 |  | 54.26 (60.89) | 50.09 (55.73) | -0.081 |
| AST (U/L), mean (SD) |  | 76.31 (130.73) | 59.95 (59.23) | -0.276 |  | 62.40 (57.47) | 62.29 (63.62) | -0.002 |
| Creatinine (μmol/L), mean (SD) |  | 66.75 (15.14) | 67.18 (17.80) | 0.024 |  | 67.68 (16.57) | 68.12 (18.50) | 0.025 |
| AFP (%) | <400 | 157 (64.1) | 76 (67.9) | 0.081 |  | 60 (65.9) | 60 (65.9) | 0.000 |
|  | ≥400 | 88 (35.9) | 36 (32.1) | -0.081 |  | 31 (34.1) | 31 (34.1) | 0.000 |
| ALBI (%) | 1 | 91 (37.1) | 32 (28.6) | -0.190 |  | 24 (26.4) | 28 (30.8) | 0.097 |
|  | ≥2 | 154 (62.9) | 80 (71.4) | 0.190 |  | 67 (73.6) | 63 (69.2) | -0.097 |

High and low refer to the CT-derived L1 attenuation groups. Abbreviations: CT, computed tomography; SD, standard deviation; BMI, body mass index; HBV, hepatitis B virus; PVTT, portal vein tumor thrombosis; BCLC, Barcelona Clinic Liver Cancer; ECOG, Eastern Cooperative Oncology Group; NLR, neutrophil-to-lymphocyte ratio; PLR, platelet-to-lymphocyte ratio; ALT, alanine aminotransferase; AST, aspartate aminotransferase; ALBI, albumin-bilirubin; AFP, alpha-fetoprotein; SMD, standardized mean difference.

## Supplementary Table 2 Tumor response in the CT-derived L1 attenuation-low and L1 attenuation-high groups before PSM

| Tumor response | L1 attenuation-low (n = 112) | L1 attenuation-high (n = 245) | *P* value |
| --- | --- | --- | --- |
| CR, n (%) | 0 (0.0%) | 0 (0.0%) |  |
| PR, n (%) | 27 (24.1%) | 80 (32.7%) |  |
| SD, n (%) | 49 (43.8%) | 108 (44.1%) |  |
| PD, n (%) | 36 (32.1%) | 57 (23.3%) |  |
| ORR (%) | 24.1% | 32.7% | 0.171 |
| DCR (%) | 67.9% | 76.7% | 0.365 |

Abbreviations: PSM, propensity score matching; CR, complete response; PR, partial response; SD, stable disease; PD, progressive disease; ORR, objective response rate; DCR, disease control rate.

## Supplementary Table 3 Tumor response in the CT-derived L1 attenuation-low and L1 attenuation-high groups after PSM

| Tumor response | L1 attenuation-low  (n = 91) | L1 attenuation-high (n = 91) | *P* value |
| --- | --- | --- | --- |
| CR, n (%) | 0 (0.0%) | 0 (0.0%) |  |
| PR, n (%) | 21 (23.1%) | 28 (30.8%) |  |
| SD, n (%) | 40 (44.0%) | 41 (45.1%) |  |
| PD, n (%) | 30 (33.0%) | 22 (24.2%) |  |
| ORR (%) | 23.1% | 30.8% | 0.317 |
| DCR (%) | 67.0% | 75.8% | 0.483 |

Abbreviations: PSM, propensity score matching; CR, complete response; PR, partial response; SD, stable disease; PD, progressive disease; ORR, objective response rate; DCR, disease control rate.

## Supplementary Table 4 Univariate and multivariable Cox proportional hazards analyses for OS before PSM

| Characteristics | Univariate analysis | | Multivariable analysis | |
| --- | --- | --- | --- | --- |
|  | Hazard ratio (95% CI) | *P* value | Hazard ratio (95% CI) | *P* value |
| Age (years) | 0.996 (0.984 - 1.008) | 0.553 |  |  |
| Sex |  |  |  |  |
| Female | Reference |  | Reference |  |
| Male | 1.672 (1.110 - 2.519) | **0.014** | 2.344 (1.508 - 3.644) | **<0.001** |
| Drinking |  |  |  |  |
| No | Reference |  |  |  |
| Yes | 0.810 (0.519 - 1.264) | 0.354 |  |  |
| HBV infection |  |  |  |  |
| No | Reference |  |  |  |
| Yes | 0.897 (0.676 - 1.192) | 0.455 |  |  |
| Tumor maximum diameter (cm) | 1.036 (1.008 - 1.065) | **0.012** | 1.004 (0.971 - 1.038) | 0.820 |
| Tumor number |  |  |  |  |
| ≤3 | Reference |  |  |  |
| >3 | 0.863 (0.596 - 1.251) | 0.437 |  |  |
| PVTT |  |  |  |  |
| No | Reference |  | Reference |  |
| Yes | 1.823 (1.380 - 2.409) | **<0.001** | 1.198 (0.838 - 1.712) | 0.321 |
| BCLC stage |  |  |  |  |
| B | Reference |  | Reference |  |
| C | 2.407 (1.758 - 3.294) | **<0.001** | 1.897 (1.252 - 2.874) | **0.003** |
| Child-Pugh |  |  |  |  |
| A | Reference |  |  |  |
| B | 1.205 (0.762 - 1.905) | 0.425 |  |  |
| ECOG |  |  |  |  |
| 0 | Reference |  |  |  |
| ≥1 | 1.197 (0.849 - 1.687) | 0.306 |  |  |
| Hepatitis drugs |  |  |  |  |
| No | Reference |  |  |  |
| Yes | 0.972 (0.704 - 1.341) | 0.862 |  |  |
| NLR | 1.037 (0.981 - 1.095) | 0.200 |  |  |
| PLR | 1.001 (0.999 - 1.003) | 0.202 |  |  |
| ALT (U/L) | 1.001 (0.999 - 1.002) | 0.342 |  |  |
| AST (U/L) | 1.000 (1.000 - 1.001) | 0.286 |  |  |
| Creatinine (μmol/L) | 0.997 (0.988 - 1.006) | 0.519 |  |  |
| ALBI |  |  |  |  |
| 1 | Reference |  |  |  |
| ≥2 | 1.280 (0.945 - 1.735) | 0.110 |  |  |
| AFP (ng/mL) |  |  |  |  |
| <400 | Reference |  | Reference |  |
| ≥400 | 2.000 (1.509 - 2.650) | **<0.001** | 1.930 (1.426 - 2.613) | **<0.001** |
| Group |  |  |  |  |
| L1 attenuation-high | Reference |  | Reference |  |
| L1 attenuation-low | 1.745 (1.286 - 2.368) | **<0.001** | 1.800 (1.308 - 2.476) | **<0.001** |

Abbreviations: OS, overall survival; PSM, propensity score matching; CI, confidence interval; HBV, hepatitis B virus; PVTT, portal vein tumor thrombosis; BCLC, Barcelona Clinic Liver Cancer; ECOG, Eastern Cooperative Oncology Group; NLR, neutrophil-to-lymphocyte ratio; PLR, platelet-to-lymphocyte ratio; ALT, alanine aminotransferase; AST, aspartate aminotransferase; ALBI, albumin-bilirubin; AFP, alpha-fetoprotein.

## Supplementary Table 5 Univariate and multivariable Cox proportional hazards analyses for PFS before PSM

| Characteristics | Univariate analysis | | Multivariable analysis | |
| --- | --- | --- | --- | --- |
|  | Hazard ratio (95% CI) | *P* value | Hazard ratio (95% CI) | *P* value |
| Age (years) | 0.989 (0.979 - 0.999) | **0.036** | 0.993 (0.980 - 1.006) | 0.284 |
| Sex |  |  |  |  |
| Female | Reference |  |  |  |
| Male | 0.887 (0.639 - 1.233) | 0.475 |  |  |
| Drinking |  |  |  |  |
| No | Reference |  |  |  |
| Yes | 1.029 (0.710 - 1.492) | 0.878 |  |  |
| HBV infection |  |  |  |  |
| No | Reference |  |  |  |
| Yes | 0.885 (0.690 - 1.135) | 0.335 |  |  |
| Tumor maximum diameter (cm) | 1.029 (1.003 - 1.055) | **0.026** | 0.993 (0.962 - 1.025) | 0.670 |
| Tumor number |  |  |  |  |
| ≤3 | Reference |  |  |  |
| >3 | 1.171 (0.840 - 1.631) | 0.352 |  |  |
| PVTT |  |  |  |  |
| No | Reference |  | Reference |  |
| Yes | 1.648 (1.283 - 2.117) | **<0.001** | 0.999 (0.719 - 1.389) | 0.996 |
| BCLC stage |  |  |  |  |
| B | Reference |  | Reference |  |
| C | 2.190 (1.697 - 2.825) | **<0.001** | 1.876 (1.337 - 2.632) | **<0.001** |
| Child-Pugh |  |  |  |  |
| A | Reference |  |  |  |
| B | 1.109 (0.747 - 1.646) | 0.609 |  |  |
| ECOG |  |  |  |  |
| 0 | Reference |  |  |  |
| ≥1 | 1.131 (0.849 - 1.507) | 0.400 |  |  |
| Hepatitis drugs |  |  |  |  |
| No | Reference |  |  |  |
| Yes | 0.800 (0.604 - 1.060) | 0.120 |  |  |
| NLR | 1.038 (0.989 - 1.089) | 0.134 |  |  |
| PLR | 1.002 (1.000 - 1.003) | **0.044** | 1.001 (0.999 - 1.003) | 0.212 |
| ALT (U/L) | 1.001 (0.999 - 1.002) | 0.447 |  |  |
| AST (U/L) | 1.000 (0.999 - 1.001) | 0.556 |  |  |
| Creatinine (μmol/L) | 0.993 (0.985 - 1.001) | **0.079** | 0.997 (0.989 - 1.006) | 0.554 |
| ALBI |  |  |  |  |
| 1 | Reference |  |  |  |
| ≥2 | 1.116 (0.859 - 1.450) | 0.409 |  |  |
| AFP (ng/mL) |  |  |  |  |
| <400 | Reference |  | Reference |  |
| ≥400 | 1.425 (1.109 - 1.831) | **0.006** | 1.136 (0.856 - 1.509) | 0.377 |
| Group |  |  |  |  |
| L1 attenuation-high | Reference |  | Reference |  |
| L1 attenuation-low | 1.447 (1.112 - 1.883) | **0.006** | 1.513 (1.112 - 2.060) | **0.008** |

Abbreviations: PFS, progression-free survival; PSM, propensity score matching; CI, confidence interval; HBV, hepatitis B virus; PVTT, portal vein tumor thrombosis; BCLC, Barcelona Clinic Liver Cancer; ECOG, Eastern Cooperative Oncology Group; NLR, neutrophil-to-lymphocyte ratio; PLR, platelet-to-lymphocyte ratio; ALT, alanine aminotransferase; AST, aspartate aminotransferase; ALBI, albumin-bilirubin; AFP, alpha-fetoprotein.

## Supplementary Table 6 Schoenfeld residual tests of the proportional hazards assumption for the multivariable OS model after PSM

| Variable | Chi-square statistic (χ²) | Degrees of freedom (df) | *P* value |
| --- | --- | --- | --- |
| Group | 2.1134 | 1 | 0.1460 |
| Sex | 1.2787 | 1 | 0.2581 |
| Tumor maximum diameter | 0.59277 | 1 | 0.4414 |
| PVTT | 0.5608 | 1 | 0.4539 |
| BCLC | 0.023046 | 1 | 0.8793 |
| AFP | 0.048257 | 1 | 0.8261 |
| ALT | 3.4118 | 1 | 0.0647 |
| AST | 1.3666 | 1 | 0.2424 |
| GLOBAL | 8.1527 | 8 | 0.4187 |

Abbreviations: OS, overall survival; PSM, propensity score matching; PVTT, portal vein tumor thrombosis; BCLC, Barcelona Clinic Liver Cancer; AFP, alpha-fetoprotein; ALT, alanine aminotransferase; AST, aspartate aminotransferase; df, degrees of freedom. Group refers to CT-derived L1 attenuation status (low versus high). GLOBAL denotes the global test of the proportional hazards assumption.

## Supplementary Table 7 Schoenfeld residual tests of the proportional hazards assumption for the multivariable PFS model after PSM

| Variable | Chi-square statistic (χ²) | Degrees of freedom (df) | *P* value |
| --- | --- | --- | --- |
| Group | 1.7249 | 1 | 0.1891 |
| Age | 0.3427 | 1 | 0.5583 |
| Sex | 0.04182 | 1 | 0.8380 |
| Tumor maximum diameter | 0.30966 | 1 | 0.5779 |
| Tumor number | 1.2988 | 1 | 0.2544 |
| PVTT | 1.0631 | 1 | 0.3025 |
| BCLC | 0.085224 | 1 | 0.7703 |
| AFP | 0.036783 | 1 | 0.8479 |
| Cre | 1.1802 | 1 | 0.2773 |
| GLOBAL | 7.737 | 9 | 0.5608 |

Abbreviations: PFS, progression-free survival; PSM, propensity score matching; PVTT, portal vein tumor thrombosis; BCLC, Barcelona Clinic Liver Cancer; AFP, alpha-fetoprotein; Cre, creatinine; df, degrees of freedom. Group refers to CT-derived L1 attenuation status (low versus high). GLOBAL denotes the global test of the proportional hazards assumption.

## Supplementary Table 8 Univariate and multivariable Cox proportional hazards analyses for overall survival after propensity score matching, including ICI type and treatment regimen.

| Characteristics | Univariate analysis | | Multivariable analysis | |
| --- | --- | --- | --- | --- |
|  | Hazard ratio (95% CI) | *P* value | Hazard ratio (95% CI) | *P* value |
| Age (years) | 0.988 (0.965 - 1.012) | 0.315 |  |  |
| Sex |  |  |  |  |
| Female | Reference |  | Reference |  |
| Male | 1.860 (1.025 - 3.376) | **0.041** | 1.980 (1.029 - 3.810) | **0.041** |
| Drinking |  |  |  |  |
| No | Reference |  |  |  |
| Yes | 0.694 (0.393 - 1.224) | 0.207 |  |  |
| HBV infection |  |  |  |  |
| No | Reference |  |  |  |
| Yes | 0.844 (0.559 - 1.273) | 0.418 |  |  |
| Tumor maximum diameter (cm) | 1.069 (1.019 - 1.121) | **0.006** | 1.020 (0.960 - 1.083) | 0.526 |
| Tumor number |  |  |  |  |
| ≤3 | Reference |  |  |  |
| >3 | 0.923 (0.551 - 1.547) | 0.762 |  |  |
| PVTT |  |  |  |  |
| No | Reference |  | Reference |  |
| Yes | 2.041 (1.377 - 3.026) | **<0.001** | 1.517 (0.897 - 2.563) | 0.120 |
| BCLC stage |  |  |  |  |
| B | Reference |  | Reference |  |
| C | 2.083 (1.326 - 3.271) | **0.001** | 1.107 (0.601 - 2.040) | 0.743 |
| Child-Pugh |  |  |  |  |
| A | Reference |  |  |  |
| B | 0.821 (0.436 - 1.547) | 0.542 |  |  |
| ECOG |  |  |  |  |
| 0 | Reference |  |  |  |
| ≥1 | 1.149 (0.749 - 1.764) | 0.525 |  |  |
| Hepatitis drugs |  |  |  |  |
| No | Reference |  |  |  |
| Yes | 1.267 (0.780 - 2.059) | 0.339 |  |  |
| NLR | 0.996 (0.923 - 1.075) | 0.913 |  |  |
| PLR | 1.000 (0.997 - 1.002) | 0.782 |  |  |
| ALT (U/L) | 1.003 (1.000 - 1.007) | **0.027** | 1.005 (0.998 - 1.012) | 0.179 |
| AST (U/L) | 1.004 (1.001 - 1.007) | **0.008** | 0.998 (0.991 - 1.005) | 0.591 |
| Creatinine (μmol/L) | 0.998 (0.986 - 1.010) | 0.778 |  |  |
| ALBI grade |  |  |  |  |
| 1 | Reference |  |  |  |
| ≥2 | 1.370 (0.867 - 2.165) | 0.177 |  |  |
| AFP (ng/mL) |  |  |  |  |
| <400 | Reference |  | Reference |  |
| ≥400 | 2.484 (1.657 - 3.723) | **<0.001** | 2.256 (1.445 - 3.524) | **<0.001** |
| Group |  |  |  |  |
| L1 attenuation-high | Reference |  | Reference |  |
| L1 attenuation-low | 1.730 (1.150 - 2.601) | **0.008** | 1.839 (1.193 - 2.833) | **0.006** |
| Treatment regimen (overall) |  | 0.206 |  |  |
| ICIs alone | Reference |  |  |  |
| ICIs + TKIs | 1.733 (1.007 - 2.981) | **0.047** |  |  |
| ICIs + VEGF | 1.234 (0.630 - 2.418) | 0.540 |  |  |
| ICIs + targeted +  interventional therapy | 1.496 (0.798 - 2.806) | 0.209 |  |  |
| Type of ICIs |  |  |  |  |
| PD-1 | Reference |  |  |  |
| PD-L1 | 0.943 (0.411 - 2.163) | 0.889 |  |  |

Abbreviations: CI, confidence interval; HBV, hepatitis B virus; PVTT, portal vein tumor thrombosis; BCLC, Barcelona Clinic Liver Cancer; ECOG, Eastern Cooperative Oncology Group; NLR, neutrophil-to-lymphocyte ratio; PLR, platelet-to-lymphocyte ratio; ALT, alanine aminotransferase; AST, aspartate aminotransferase; ALBI, albumin-bilirubin; AFP, alpha-fetoprotein; ICIs, immune checkpoint inhibitors; TKIs, tyrosine kinase inhibitors; VEGF, vascular endothelial growth factor; PD-1, programmed cell death protein 1; PD-L1, programmed death-ligand 1.

## Supplementary Table 9 Univariate and multivariable Cox proportional hazards analyses for progression-free survival after propensity score matching, including ICI type and treatment regimen.

| Characteristics | Univariate analysis | | Multivariable analysis | |
| --- | --- | --- | --- | --- |
|  | Hazard ratio (95% CI) | *P* value | Hazard ratio (95% CI) | *P* value |
| Age (years) | 0.974 (0.957 - 0.991) | **0.004** | 0.991 (0.968 - 1.014) | 0.442 |
| Sex |  |  |  |  |
| Female | Reference |  | Reference |  |
| Male | 0.578 (0.365 - 0.916) | **0.020** | 0.677 (0.392 - 1.172) | 0.164 |
| Drinking |  |  |  |  |
| No | Reference |  |  |  |
| Yes | 1.006 (0.630 - 1.605) | 0.981 |  |  |
| HBV infection |  |  |  |  |
| No | Reference |  |  |  |
| Yes | 0.779 (0.545 - 1.112) | 0.169 |  |  |
| Tumor maximum diameter (cm) | 1.068 (1.024 - 1.114) | **0.002** | 1.035 (0.983 - 1.089) | 0.190 |
| Tumor number |  |  |  |  |
| ≤3 | Reference |  | Reference |  |
| >3 | 1.812 (1.149 - 2.857) | **0.011** | 1.553 (0.921 - 2.620) | 0.099 |
| PVTT |  |  |  |  |
| No | Reference |  | Reference |  |
| Yes | 2.041 (1.435 - 2.902) | **<0.001** | 1.149 (0.715 - 1.848) | 0.565 |
| BCLC stage |  |  |  |  |
| B | Reference |  | Reference |  |
| C | 2.494 (1.730 - 3.595) | **<0.001** | 1.644 (0.994 - 2.720) | 0.053 |
| Child-Pugh |  |  |  |  |
| A | Reference |  |  |  |
| B | 0.784 (0.470 - 1.308) | 0.351 |  |  |
| ECOG |  |  |  |  |
| 0 | Reference |  |  |  |
| ≥1 | 1.004 (0.693 - 1.455) | 0.984 |  |  |
| Hepatitis drugs |  |  |  |  |
| No | Reference |  |  |  |
| Yes | 0.862 (0.565 - 1.315) | 0.491 |  |  |
| NLR | 0.991 (0.928 - 1.058) | 0.789 |  |  |
| PLR | 1.001 (0.998 - 1.003) | 0.637 |  |  |
| ALT (U/L) | 1.000 (0.997 - 1.003) | 0.897 |  |  |
| AST (U/L) | 1.001 (0.998 - 1.004) | 0.505 |  |  |
| Creatinine (μmol/L) | 0.984 (0.972 - 0.995) | **0.007** | 0.996 (0.984 - 1.009) | 0.544 |
| ALBI grade |  |  |  |  |
| 1 | Reference |  |  |  |
| ≥2 | 1.054 (0.720 - 1.542) | 0.786 |  |  |
| AFP (ng/mL) |  |  |  |  |
| <400 | Reference |  | Reference |  |
| ≥400 | 1.837 (1.285 - 2.626) | **<0.001** | 1.339 (0.896 - 2.000) | 0.154 |
| Group |  |  |  |  |
| L1 attenuation-high | Reference |  | Reference |  |
| L1 attenuation-low | 1.471 (1.039 - 2.083) | **0.030** | 1.458 (1.007 - 2.110) | **0.046** |
| Treatment regimen (overall) |  | **0.009** |  | 0.117 |
| ICIs alone | Reference |  | Reference |  |
| ICIs + TKIs | 1.899 (1.194 - 3.020) | **0.007** | 1.359 (0.818 - 2.255) | 0.236 |
| ICIs + VEGF | 1.571 (0.905 - 2.728) | 0.108 | 1.059 (0.585 - 1.918) | 0.850 |
| ICIs + targeted +  interventional therapy | 2.338 (1.360 - 4.021) | **0.002** | 1.951 (1.088 - 3.498) | **0.025** |
| Type of ICIs |  |  |  |  |
| PD-1 | Reference |  |  |  |
| PD-L1 | 0.658 (0.303 - 1.431) | 0.291 |  |  |

Abbreviations: CI, confidence interval; HBV, hepatitis B virus; PVTT, portal vein tumor thrombosis; BCLC, Barcelona Clinic Liver Cancer; ECOG, Eastern Cooperative Oncology Group; NLR, neutrophil-to-lymphocyte ratio; PLR, platelet-to-lymphocyte ratio; ALT, alanine aminotransferase; AST, aspartate aminotransferase; ALBI, albumin-bilirubin; AFP, alpha-fetoprotein; ICIs, immune checkpoint inhibitors; TKIs, tyrosine kinase inhibitors; VEGF, vascular endothelial growth factor; PD-1, programmed cell death protein 1; PD-L1, programmed death-ligand 1.

## Supplementary Table 10 Correlation coefficients and P values among L1 attenuation group status and clinical characteristics

|  | Age | BMI | Hb | Child-Pugh | ALBI | Cirrhosis | ECOG | NLR | PLR | Group |
| --- | --- | --- | --- | --- | --- | --- | --- | --- | --- | --- |
| Age |  | 0.091 | 0.044 | 0.594 | 0.233 | 0.299 | 0.013 | 0.906 | 0.007 | <0.001 |
| BMI | ρ = 0.090 |  | <0.001 | 0.890 | 0.023 | 0.439 | 0.117 | 0.469 | 0.310 | 0.455 |
| Hb | ρ = -0.109 | ρ = 0.226 |  | 0.472 | <0.001 | 0.574 | 0.923 | <0.001 | 0.001 | 0.012 |
| Child-Pugh | ρ = 0.028 | ρ = -0.007 | ρ = -0.039 |  | 0.029 | 0.026 | 1.000 | 0.069 | 0.422 | 0.017 |
| ALBI | ρ = 0.064 | ρ = -0.122 | ρ = -0.263 | ρ = 0.125 |  | 0.017 | 0.298 | 0.432 | 0.246 | 0.185 |
| Cirrhosis | ρ = 0.056 | ρ = 0.041 | ρ = -0.030 | ρ = 0.122 | ρ = 0.130 |  | 0.210 | 0.324 | 0.011 | 0.492 |
| ECOG | ρ = 0.132 | ρ = -0.083 | ρ = -0.005 | ρ = -0.009 | ρ = 0.059 | ρ = -0.070 |  | 0.179 | 0.454 | 0.184 |
| NLR | ρ = 0.006 | ρ = -0.039 | ρ = -0.221 | ρ = 0.097 | ρ = 0.042 | ρ = -0.053 | ρ = 0.072 |  | <0.001 | 0.036 |
| PLR | ρ = -0.143 | ρ = -0.054 | ρ = -0.171 | ρ = 0.043 | ρ = -0.063 | ρ = -0.137 | ρ = -0.041 | ρ = 0.562 |  | 0.922 |
| Group | ρ = 0.346 | ρ = 0.040 | ρ = -0.134 | ρ = 0.130 | ρ = 0.078 | ρ = 0.038 | ρ = 0.071 | ρ = 0.112 | ρ = 0.005 |  |

The lower triangle shows Spearman correlation coefficients (ρ), and the upper triangle shows statistical P values. BMI, body mass index; Hb, hemoglobin; ALBI, albumin–bilirubin category; ECOG, Eastern Cooperative Oncology Group performance status; NLR, neutrophil-to-lymphocyte ratio; PLR, platelet-to-lymphocyte ratio.

# Supplementary Figures

## Supplementary Figure 1. The optimal cutoff value for CT-derived L1 attenuation classification was established by the X-tile software. (A) X-tile plots. (B) Histograms.

## Supplementary Figure 2. Love plot showing baseline covariate balance before and after propensity score matching.

Red and turquoise dots represent the absolute standardized mean differences before matching (unmatched) and after matching (matched), respectively. The vertical dashed line indicates an absolute standardized mean difference of 0.1. Smaller values indicate better balance between the CT-derived L1 attenuation-low and attenuation-high groups. SMD, standardized mean difference; BMI, body mass index; HBV, hepatitis B virus; PVTT, portal vein tumor thrombosis; BCLC, Barcelona Clinic Liver Cancer; ECOG, Eastern Cooperative Oncology Group; NLR, neutrophil-to-lymphocyte ratio; PLR, platelet-to-lymphocyte ratio; ALT, alanine aminotransferase; AST, aspartate aminotransferase; ALBI, albumin-bilirubin; AFP, alpha-fetoprotein.

## Supplementary Figure 3. Bootstrap assessment (A) of cutoff stability and restricted cubic spline analysis (B) of the association between CT-derived vertebral attenuation and OS.

(A) Distribution of OS cutoffs reselected in 1,000 bootstrap resamples. The orange dashed line indicates 100 HU, and the black dotted line indicates the full-cohort cutoff of 100.5 HU. (B) The solid blue curve represents the adjusted HR, with the shaded area indicating the confidence interval. The vertical dotted line indicates the reference value of 100 HU, and the horizontal dashed line indicates an HR of 1. Rug marks along the x-axis show the distribution of observed vertebral attenuation values. OS, overall survival; HR, hazard ratio; HU, Hounsfield units.

## Supplementary Figure 4. Scatter plot showing the correlation between CT-derived L1 attenuation and corresponding DXA T-scores.

Red dots represent patients with osteoporosis (T-score ≤ -2.5); yellow dots represent patients with osteopenia (-2.5 < T-score < -1); green dots represent patients with normal bone density (T-score ≥ -1). DXA, dual-energy X-ray absorptiometry.

## Supplementary Figure 5. Forest plot of subgroup analyses for overall survival after propensity score matching, including ICI type and treatment regimen.

Points represent HRs, and horizontal lines indicate 95% CIs. The vertical dashed line denotes an HR of 1, and the horizontal axis is logarithmic. Arrowheads indicate confidence limits beyond the plotted range. P for int., P value for interaction; HR, hazard ratio; CI, confidence interval; HBV, hepatitis B virus; ECOG, Eastern Cooperative Oncology Group; PVTT, portal vein tumor thrombosis; BCLC, Barcelona Clinic Liver Cancer; AFP, alpha-fetoprotein; ALBI, albumin-bilirubin; ICIs, immune checkpoint inhibitors; TKIs, tyrosine kinase inhibitors; VEGF, vascular endothelial growth factor; PD-1, programmed cell death protein 1; PD-L1, programmed death-ligand 1.

## Supplementary Figure 6. Forest plot of subgroup analyses for progression-free survival after propensity score matching, including ICI type and treatment regimen.

Points represent HRs, and horizontal lines indicate 95% CIs. The vertical dashed line denotes an HR of 1, and the horizontal axis is logarithmic. Arrowheads indicate confidence limits beyond the plotted range. P for int., P value for interaction; HR, hazard ratio; CI, confidence interval; HBV, hepatitis B virus; ECOG, Eastern Cooperative Oncology Group; PVTT, portal vein tumor thrombosis; BCLC, Barcelona Clinic Liver Cancer; AFP, alpha-fetoprotein; ALBI, albumin-bilirubin; ICIs, immune checkpoint inhibitors; TKIs, tyrosine kinase inhibitors; VEGF, vascular endothelial growth factor; PD-1, programmed cell death protein 1; PD-L1, programmed death-ligand 1.

## Supplementary Figure 7. Heat map of correlation coefficients between L1 attenuation group status and selected clinical characteristics.

BMI, body mass index; Hb, hemoglobin; ALBI, albumin–bilirubin category; ECOG, Eastern Cooperative Oncology Group; NLR, neutrophil-to-lymphocyte ratio; PLR, platelet-to-lymphocyte ratio.
